# A Matched Case-Control Study of Risk Factors for Breast Cancer Risk in Vietnam

**DOI:** 10.1155/2016/7164623

**Published:** 2016-12-13

**Authors:** J. Nguyen, Q. H. Le, B. H. Duong, P. Sun, H. T. Pham, V. T. Ta, J. Kotsopoulos, S. A. Narod, O. Ginsburg

**Affiliations:** ^1^Queen's University, Kingston, ON, Canada; ^2^Lakeridge Health, Oshawa, ON, Canada; ^3^Hospital K, National Cancer Hospital of Vietnam, Hanoi, Vietnam; ^4^Women's College Research Institute, Women's College Hospital, Toronto, ON, Canada; ^5^Dalla Lana School of Public Health, University of Toronto, Toronto, ON, Canada

## Abstract

*Background*. Vietnam has a low age-standardized incidence of breast cancer, but the incidence is rising rapidly with economic development. We report data from a matched case-control study of risk factors for breast cancer in the largest cancer hospital in Vietnam.* Methods*. 492 incident breast cancer cases unselected for family history or age at diagnosis and 1306 control women age 25–75 were recruited from the National Cancer Hospital (BVK), Hanoi. Structured interviews were conducted and pathology data was centrally reported at the National Cancer Hospital of Vietnam, in Hanoi.* Results*. Our analysis included 294 matched pairs. Mean age at diagnosis was 46.7 years. Lower mean parity, older age at first parity, increasing weight and BMI at age 18, and increasing BMI at diagnosis were positively correlated with breast cancer cases compared to controls. Age at first menarche and duration of breastfeeding were not statistically different between cases and controls.* Conclusions*. In this study we demonstrate that breast cancer in Vietnam is associated with some but not all of the published risk factors from Western populations. Our data is consistent with other studies of breast cancer in Asian populations.

## 1. Introduction

The incidence of breast cancer in developing countries is increasing, and low and middle income countries now account for most deaths from breast cancer globally [1]. The age-standardized risk of developing breast cancer varies considerably across populations [1], and the risk is predicted to increase in countries undergoing rapid development [2].

Several hormonal and reproductive risk factors have been implicated in the development of breast cancer in women of European ancestry, but it is not clear to what extent these factors contribute to breast cancer in Asia. These include reproductive (early age at menarche, nulliparity, later age at first childbirth, lack of breastfeeding, later age at menopause, and the use of combined hormone replacement therapy) and anthropometric risk factors (height, weight, and body mass index (BMI)) [3–7]. The impact of breast cancer risk factors varies depending on ethnicity and geographic region [8–14].

Vietnam has a population of 87 million and reports one of the lowest age-standardized incidence rates of breast cancer in the world, at 15–27 cases per 100,000 per year [15].

In the current study, we sought to identify hormonal, reproductive, and anthropometric risk factors for both pre- and postmenopausal breast cancer in Vietnamese women.

## 2. Methods

### 2.1. Study Design

This single-center, hospital-based, case-control study was conducted between January 2007 and August 2013 at the National Cancer Hospital of Vietnam in Hanoi, Vietnam. This is the largest tertiary care cancer hospital in Vietnam and is also the site of the National Institute for Cancer Control.

### 2.2. Cases

We defined a case as a woman aged 25 to 75 years of age, who was diagnosed with invasive breast cancer in the 12 months prior to study enrollment. Cases were recruited from the inpatient and outpatient departments of the National Cancer Hospital of Vietnam, in Hanoi.

Women who had a previous history of breast cancer (in-situ or invasive) were not eligible for this study. Women were considered to be premenopausal if they had reported a menstrual period within the 12 months prior to their breast cancer diagnosis. Women who had not experienced a menstrual period for 12 consecutive months or women who had a hysterectomy (with or without) oophorectomy were considered postmenopausal.

For the 492 cases of breast cancer, pathology data was systematically recorded at the National Cancer Hospital. Data collected included histopathological type, Nottingham grade, TNM stage, and ER/PR/Her2 status. Immunohistochemistry for ER/PR/Her2 was performed on-site. Her2 testing was performed by immunohistochemistry alone.

### 2.3. Controls

A potential control was a woman aged 25 years or older who had no prior history of breast cancer and who was unrelated to cases. Controls were recruited from a variety of settings, including outpatient clinics at the National Cancer Hospital of Vietnam and from rural primary care clinics. The National Cancer Hospital is located in Hanoi, the metropolitan area which has approximately 7 million citizens. Controls were also purposefully recruited from rural areas, where epidemiological surveillance for many health conditions is routinely undertaken and from where many women with breast cancer at the National Cancer Hospital would likely come from.

As urban residence is a recognized risk factor for breast cancer in other populations studied, we purposefully sampled controls from both rural and urban areas and then matched cases with controls on this basis, as described below.

### 2.4. Matching

Cases and controls were matched by date of birth within one year, place of birth (rural or urban), and place of current residence (rural or urban).

### 2.5. Data Collection

Written informed consent was obtained, and structured interviews were conducted by a single Vietnamese research associate based at the National Cancer Institute and included a questionnaire regarding demographic, hormonal, reproductive, and anthropometric factors. Specifically, the questionnaire asked about date of birth, income, education, age at menarche, age at first childbirth, total number of children, duration of breast feeding (in months), current height (meters), weight at the age 18 years (kg), and weight at the time of breast cancer diagnosis (or at interview, for controls). For each study subject we calculated body mass index (BMI) by the weight in kilograms divided by the square of the height in meters (kg/m^2^). We defined four categories of BMI according to the World Health Organization classification [16]: underweight < 18.50 kg/m^2^; normal range 18.50 to 24.99 mg/m^2^; overweight ≥ 25 mg/m^2^; and obese ≥ 30 mg/m^2^. For both cases and controls, we relied on self-reported values of weight in order to calculate the BMI at the age 18 years. Although there have been attempts to tailor BMI categories for Asian populations, the World Health Organization recommends that the current cut-off points should be retained as the international classification.

### 2.6. Ethical Considerations

The study was approved by the Research Ethics Board of Queens University (Kingston, Ontario, Canada) and the Research Ethics Committee and administration of the National Cancer Hospital, Hanoi.

### 2.7. Statistical Analysis

Conditional logistic regression analysis was performed to evaluate the association of various risk factors with the risk of breast cancer, displayed as odds ratios, OR with 95% confidence intervals. A *p* < 0.05 was considered statistically significant. All *p* values were two-tailed.

## 3. Results

### 3.1. Clinical Features and Pathological Data

The clinical features and pathological data for cases are shown in Table 1. The mean age of breast cancer diagnosis was 45.9 years (range 24–65). The majority of patients were both born and raised in cities in Vietnam. The majority of the breast cancer patients (61.6%) were ER-positive, 58.5% of cases were PR positive, and 21.4% of cases were Her2 positive. Fifteen percent were triple negative (ER negative, PR negative, and Her2 negative).

### 3.2. Hormonal, Reproductive, and Anthropometric Risk Factors

The distributions of the hormonal, reproductive, and anthropometric characteristics of the 294 breast cancer cases and their matched controls are presented in Table 2. Compared to controls, cases had greater weight at age 18 (47.2 versus 46.2 kg, *p* = 0.03), a higher BMI at age 18 (19.7 versus 19.1 kg/m^2^, *p* = 0.002), and a higher BMI at the date of diagnosis (21.4 versus 20.8 kg/m^2^, *p* = 0.002).

Breast cancer cases had a significantly lower mean parity (2.3 versus 2.6 children, *p* < 0.001) and older age at first childbirth (24.5 versus 23.8 years, *p* = 0.05). There were no significant differences seen between cases and controls for the other reproductive factors including age at menarche (15.8 versus 16.0 years, *p* = 0.2), breastfeeding (total duration 35.7 versus 37.6 months, *p* = 0.38), height (154.8 cm versus 155.4 cm, *p* = 0.1), or menopausal status (25.5 versus 23.8% menopausal, *p* = 0.64).  Table 3 shows the unadjusted (univariate) and adjusted (multivariate) odds ratios and 95% confidence intervals for breast cancer.

Body mass index was positively associated with breast cancer risk. Compared to women with a BMI less than 18 kg/m^2^ at age 18, those with a BMI greater than 20 kg/m^2^ at age 18 were associated with a significantly increased risk of breast cancer after adjusting for other factors (adjusted OR = 1.72, 95% CI 1.07–2.76, *p* = 0.003).

The most profound risk factor was parity. Compared to nulliparous women, increasing parity was associated with a significantly decreased risk of breast cancer; that is, for four or more births, the unadjusted odds ratio was 0.16 (95% CI 0.04–0.55, *p* = 0.004) and the adjusted odds ratio was 0.17 (95% CI 0.05–0.63, *p* = 0.008). Age at menarche, age at first parity, total months breastfeeding, oral contraceptive use, and menopause were not associated with increased breast cancer risk in this analysis.

We performed subgroup analysis for premenopausal cases (196 cases and 196 matched controls) (Tables 4 and 5). Among premenopausal cases we observed strong associations for weight at age 18 (47.4 versus 45.8 kg; *p* < 0.002) and BMI at age 18 (19.7 versus 18.8 kg/m^2^, *p* < 0.0001) for cases compared to controls. Age at first birth was not associated with the risk of premenopausal breast cancer (24.2 versus 23.7 years, *p* < 0.25).

We also performed the same analysis for ER-positive cases only. However, the odds ratios did not vary significantly from those in the whole dataset (data not shown).

Lastly, although WHO does not recommend a revised international standard for BMI categories, some reports suggest a tailored evaluation for individual Asian populations. We therefore reclassified BMI groups by the frequency of BMI 18 for cases, at 33.33% and 66.66%. The odds ratio results were very close to our original classification; for example, BMI > 20.46 (rather than >20.00) yielded a multivariate OR 1.73 (*p* = 0.004), compared with 1.72 (*p* = 0.003).

## 4. Discussion

In this study we evaluate the role of hormonal, reproductive, and anthropometric risk factors of the risk of breast cancer in Vietnamese women. While some of our findings are consistent with those in studies of Western populations, other such associations were not identified in our dataset.

Lower parity and older age at first birth were both risk factors for breast cancer. These are well-established risk factors in Western women [3, 7, 17, 18]. In contrast, neither age at menarche nor duration of breastfeeding were found to be associated with breast cancer risk in our dataset. The associations with hormonal and reproductive risk factors were not dependent on the ER-status of the cases. In Western populations, some studies suggest that these factors are more strongly correlated with ER-positive breast cancers [19–21].

### 4.1. Anthropometric Risk Factors

Greater weight at age 18, BMI at age 18, and BMI at diagnosis were positively correlated with breast cancer in this study. This trend was strengthened by selection for premenopausal cases only. This differs from studies in Western populations, where overweight or obesity at age 18, increased BMI at age 18, and BMI at diagnosis have been associated with a lower risk of premenopausal breast cancer [3–6]. However, it should be noted that these studies in Western populations used BMI > 25 or >30 to define overweight/obesity, while there were very few Vietnamese women in our study that would be characterized as overweight/obese based on BMI (mean BMI was 19.7 kg/m^2^, range 14.7–26.3).

Our findings agree with a meta-analysis of cohort studies that showed that breast cancer risk was increased by 16% per 5 kg/m^2^ increment of BMI in premenopausal Asian women but decreased by 9% per 5 kg/m^2^ increment of BMI in premenopausal North American women [22].

### 4.2. Comparison to Other Studies in Vietnam

Nichols and colleagues performed a risk factor analysis of premenopausal breast cancer in Vietnam [23]. They also reported lower mean parity and increasing age at first parity as positively associated with breast cancer; however, they did not find weight or BMI to be correlated with breast cancer risk. The reason for these differences between our study and the study by Nichols et al. is unclear, but it may be related to differences in study design. Our study recruited both pre- and postmenopausal women and matched based on place of birth, place of current residence, and age, whereas their study only recruited premenopausal women and did not match for place of birth or place of current residence (rural/urban).

### 4.3. Comparison to Studies of Different Ethnicities

Breast cancer epidemiological studies in other Asian countries suggest that breast cancer risk factors vary with ethnicity. A cohort study of 4211 Chinese women demonstrated that family history, increased BMI, and later menopause were strongly correlated with breast cancer risk [9]. However, other risk factors observed in Western populations, such as nulliparity and no history of breastfeeding, were not observed in this Chinese population. In contrast, Iwasaki and Tsugane reported that early age at menarche, nulliparity, low parity, and late age at first birth were correlated with breast cancer risk in a Japanese case-control study [10]. However, other risk factors in Western populations, such as no history of breastfeeding, were not observed in this Japanese population.

Taken together, our study is in keeping with others from Asia, which demonstrates that breast cancer risk factors vary across different ethnic groups [8–14, 24–26].

### 4.4. Study Limitations

Our study has several limitations. Study participants were asked to recall their weight (and thus body mass index) at age 18. Controls were also asked to recall their current weight.

However, there is no reason to suspect a systemic bias in recalling weigh between cases and controls. Very few women from either group were able to report a family history of cancer (data not shown). This may be for social and/or cultural reasons, which have been suggested as a cause of underreporting of cancer family histories in other Asian studies including our previous study on family history and BRCA 1 and 2 mutations in Vietnam [27, 28].

Our study was underpowered to examine risk factors among specific breast cancer subtypes, such as ER negative breast cancer (*n* = 93) and triple negative breast cancer (*n* = 44). This is important because studies in Western populations have demonstrated that breast cancer is highly heterogeneous and risk factors may differ between different breast cancer subtypes [19–21, 29, 30]. This study is also likely underpowered to draw conclusions about whether OCP use and HRT use are breast cancer risk factors in Vietnamese women, because the use of OCP and HRT in Vietnam has traditionally been much lower than in Western populations.

## 5. Conclusions

Breast cancer is a heterogeneous disease with variations in the prevalence of risk factors across ethnicities. In this study we demonstrate that breast cancer in Vietnam is associated with some but not all of the published risk factors from Western populations. Our data is consistent with other studies of breast cancer in Asian populations. This has potential implications for education and health promotion about breast cancer, as well as risk assessment tools of relevant women in Vietnam, where the incidence of breast cancer continues to increase annually.

## Figures and Tables

**Table 1:** Clinical features and pathological data for cases.

Feature	Result	Cases (*n* = 294)	% of Total Cases
Estrogen receptor (ER)	Positive	181	61.6
	Negative	93	31.6
	Unavailable	20	6.8

Progesterone receptor (PR)	Positive	172	58.5
	Negative	102	34.7
	Unavailable	20	6.8

Her2/neu	Positive	63	21.4
	Negative	209	71.1
	Unavailable	22	7.5

Pathological type	Ductal carcinoma in situ	3	1.0
	Invasive ductal carcinoma	205	69.7
	Invasive lobular carcinoma	15	5.1
	Mucinous carcinoma	8	2.7
	Papillary carcinoma	10	3.4
	Tubular carcinoma	8	2.7
	Unavailable	45	15.3

Histological Grade^*∗*^	1	25	8.5
	2	129	43.9
	3	49	16.7
	Unavailable	91	31.0

Tumour (T) stage	1	20	6.8
	2	199	67.7
	3	18	6.1
	4	10	3.4
	Unavailable	47	16.0

Node (N) stage	0	111	37.8
	1	97	33.0
	2	39	13.3
	Unavailable	47	16.0

Metastasis (M) stage	0	246	83.7
	1	1	0.3
	Unavailable	47	16.0

AJCC clinical Stage^*∗∗*^	1	13	4.4
	2a	102	34.7
	2b	77	26.2
	3a	40	13.6
	3b	14	4.8
	4	1	0.3
	Unavailable	47	16.0

^*∗*^Graded based on the Nottingham grading system.

*∗∗* refers to American Joint Committee on Cancer (AJCC) staging system for breast cancer.

**Table 2:** Characteristics of breast cancer cases and matched controls.

Risk factors	Characteristics	Cases (*n* = 294)	Controls (*n* = 294)	*p*
Reproductive factors	Age at menarche (years)	15.8	16	0.2
	Parity			
	0	14 (4.9%)	5 (1.7%)	
	1	29 (9.8%)	17 (5.8%)	
	2	150 (51.0%)	123 (41.8%)	
	3	62 (21.1%)	98 (33.3%)	
	4+	30 (10.2%)	49 (16.7%)	
	Mean parity	2.3	2.6	<0.0001
	Age at first parity (years)	24.5	23.8	0.05
	Total months breastfeeding	35.7	37.6	0.38

Anthropometric factors	Height (cm)^*∗*^	154.8	155.4	0.1
	Weight at age 18 (kg)	47.2	46.2	0.03
	BMI at age 18	19.7	19.1	0.002
	BMI gain since age 18	1.72	1.66	0.82
	BMI at diagnosis/interview^*∗*^	21.4	20.8	0.002
	BMI at age 18			
	<18 kg/m^2^	65 (22.2%)	75 (26.2%)	
	18–20 kg/m^2^	98 (33.6%)	124 (43.4%)	
	>20 kg/m^2^	130 (44.4%)	87 (30.4%)	

Hormonal factors	Oral contraceptive use			
	No	266 (91.4%)	278 (95.5%)	
	Yes	25 (8.6%)	13 (4.5%)	0.04
	Menopause			
	No	196 (74.5%)	218 (76.2%)	
	Yes	67 (25.5%)	68 (23.8%)	0.64

^*∗*^For cases refers to BMI at diagnosis of breast cancer, prior to treatment. For controls, it refers to BMI at time of interview.

**Table 3:** Conditional logistic regression analysis to estimate the odds (odd ratio, OR) of breast cancer.

	Cases	Controls	Unadjusted OR			Adjusted OR		
			OR	95% CI	*p*	OR	95% CI	*p*
*Reproductive factors*								
Age at menarche (years)^*∗*^	15.8	16	0.94	0.86–1.03	0.16	0.95	0.86–1.05	0.28
Parity^*∗*^								
0	14 (4.9%)	5 (1.7%)	1			1		
1	29 (9.8%)	17 (5.8%)	0.5	0.15–1.72	0.27	0.5093	0.14–1.75	0.19
2	150 (51.0%)	123 (41.8%)	0.34	0.11–1.10	0.07	0.33	0.10–1.09	0.81
3	62 (21.1%)	98 (33.3%)	0.18	0.05–0.58	0.004	0.17	0.05–0.59	0.001
4+	30 (10.2%)	49 (16.7%)	0.16	0.04–0.55	0.004	0.17	0.05–0.63	0.008
Age at 1st parity	24.5	23.8	1.05	1.00–1.10	0.06			
Total months breastfeeding	35.7	37.6	1	0.99–1.02	0.91			

*Anthropometric factors*								
BMI at age 18^*∗*^								
<18 kg/m^2^	65 (22.2%)	75 (26.2%)	1			1		
18–20 kg/m^2^	98 (33.6%)	124 (43.4%)	0.9188	0.59–1.38	0.36	1.0072	0.63–1.57	0.23
>20 kg/m^2^	130 (44.4%)	87 (30.4%)	1.79	1.16–2.78	0.0011	1.72	1.07–2.76	0.003

*Hormonal factors*								
OCP Use^*∗*^								
No	266 (91.4%)	278 (95.5%)	1			1		
Yes	25 (8.6%)	13 (4.5%)	2	1.00–4.00	0.05	2.03	0.94–4.42	0.07
Menopause								
No	196 (74.5%)	218 (76.2%)	1					
Yes	67 (25.5%)	68 (23.8%)	1.04	0.61–1.76	0.89			

*∗* denotes the variables used for multivariate analysis to calculate the adjusted OR.

**Table 4:** Characteristics of breast cancer cases and matched controls for premenopausal women.

Risk factors	Characteristics	Cases (*n* = 196)	Controls (*n* = 196)	*p*
Reproductive factor	Age at menarche (years)	15.8	15.7	0.64
	Parity			
	0	10 (5.3%)	4 (2.1%)	
	1	16 (8.2%)	7 (3.6%)	
	2	110 (56.1%)	96 (49.0%)	
	3	39 (19.9%)	64 (32.7%)	
	4+	14 (7.1%)	24 (12.2%)	
	Mean parity	2.2	2.5	0.0002
	Age at first parity (years)	24.2	23.7	0.25
	Total months breastfeeding	36.1	37.4	0.65

Anthropometric factors	Height (cm)^*∗*^	155.1	155.9	0.1
	Weight at age 18 (kg)	47.4	45.8	0.002
	BMI at age 18	19.7	18.8	0.0001
	BMI gain since age 18	1.69	1.84	0.60
	BMI at diagnosis/interview^*∗*^	21.44	21.68	0.002
	BMI at age 18			
	<18 kg/m^2^	42 (21.4%)	60 (30.6%)	
	18–20 kg/m^2^	64 (32.7%)	80 (40.8%)	
	>20 kg/m^2^	89 (45.4%)	50 (25.5%)	

Hormonal factors	Oral contraceptive use			
	No	173 (89.2%)	184 (94.9%)	
	Yes	21 (10.8%)	10 (5.2%)	0.04

^*∗*^For cases refers to BMI at diagnosis of breast cancer, prior to treatment. For controls, it refers to BMI at time of interview.

**Table 5:** Conditional logistic regression analysis to estimate the odds (odd ratio, OR) of breast cancer in premenopausal women.

	Cases	Controls	Unadjusted OR			Adjusted OR		
			OR	95% CI	*p*	OR	95% CI	*p*
*Reproductive factors*								
Age at menarche (years)^*∗*^	15.8	15.7	1.03	0.92–1.14	0.64	1	0.89–1.15	0.84
Parity^*∗*^								
0	10 (5.3%)	4 (2.1%)	1			1		
1	16 (8.2%)	7 (3.6%)	0.83	0.17–4.05	0.82	1.25	0.24–6.63	0.05
2	110 (56.1%)	96 (49.0%)	0.38	0.10–1.47	0.16	0.43	0.11–1.72	0.47
3	39 (19.9%)	64 (32.7%)	0.19	0.05–0.74	0.02	0.22	0.05–0.95	0.004
4+	14 (7.1%)	24 (12.2%)	0.25	0.06–1.09	0.06	0.28	0.06–1.26	0.09
Age at 1st parity	24.2	23.7	1.03	0.97–1.09	0.33			
Total months breastfeeding	36.1	37.4	1	0.99–1.02	0.81			

*Anthropometric factors*								
BMI at age 18^*∗*^								
<18 kg/m^2^	42 (21.5%)	60 (31.6%)	1			1		
18–20 kg/m^2^	64 (32.8%)	80 (42.1%)	1.16	0.69–1.94	0.44	1.33	0.76–2.34	0.24
>20 kg/m^2^	89 (45.6%)	50 (26.3%)	2.73	1.56–4.79	0.0006	2.801	1.51–5.20	0.001

*Hormonal factors*								
OCP Use^*∗*^								
No	173 (89.2%)	184 (94.9%)	1			1		
Yes	21 (10.8%)	10 (5.2%)	2.2	1.01–4.89	0.05	2.11	0.89–5.01	0.09

*∗* denotes the variables used for multivariate analysis to calculate the adjusted OR.

## References

[1] Ferlay J., Soerjomataram I., Ervik M. (2013). *GLOBOCAN 2012 v1.0, Cancer Incidence and Mortality Worldwide: IARC Cancer Base No. 11*.

[2] Bray F., Jemal A., Grey N., Ferlay J., Forman D. (2012). Global cancer transitions according to the human development index (2008–2030): a population-based study. *The Lancet Oncology*.

[3] Nelson H. D., Zakher B., Cantor A. (2012). Risk factors for breast cancer for women aged 40 to 49 years: a systematic review and meta-analysis. *Annals of Internal Medicine*.

[4] Huang Z., Hankinson S. E., Colditz G. A. (1997). Dual effects of weight and weight gain on breast cancer risk. *Journal of the American Medical Association*.

[5] van den Brandt P. A., Spiegelman D., Yaun S.-S. (2000). Pooled analysis of prospective cohort studies on height, weight, and breast cancer risk. *American Journal of Epidemiology*.

[6] Reeves G. K., Pirie K., Beral V., Green J., Spencer E., Bull D. (2007). Cancer incidence and mortality in relation to body mass index in the Million Women Study: cohort study. *British Medical Journal*.

[7] Rosner B., Colditz G. A., Willett W. C. (1994). Reproductive risk factors in a prospective study of breast cancer: the nurses' health study. *American Journal of Epidemiology*.

[8] Yuan J.-M., Yu M. C., Ross R. K., Gao Y.-T., Henderson B. E. (1988). Risk factors for breast cancer in Chinese women in Shanghai. *Cancer Research*.

[9] Lee H., Li J.-Y., Fan J.-H. (2014). Risk factors for breast cancer among chinese women: a 10-year nationwide multicenter cross-sectional study. *Journal of Epidemiology*.

[10] Iwasaki M., Tsugane S. (2011). Risk factors for breast cancer: epidemiological evidence from Japanese studies. *Cancer Science*.

[11] Howlader N., Noone A. M., Krapcho M. (1975). *SEER Cancer Statistics Review, 1975–2011*.

[12] Chlebowski R. T., Chen Z., Anderson G. L. (2005). Ethnicity and breast cancer: factors influencing differences in incidence and outcome. *Journal of the National Cancer Institute*.

[13] Krieger N., Chen J. T., Waterman P. D. (2010). Decline in US breast cancer rates after the women's health initiative: socioeconomic and racial/ethnic differentials. *American Journal of Public Health*.

[14] Gilliland F. D., Hunt W. C., Baumgartner K. B. (1998). Reproductive risk factors for breast cancer in Hispanic and non-Hispanic white women: the New Mexico Women's Health Study. *American Journal of Epidemiology*.

[15] Anh P. T. H., Duc N. B. (2002). The situation with cancer control in Vietnam. *Japanese Journal of Clinical Oncology*.

[16] World Health Organization BMI classification http://apps.who.int/bmi/index.jsp?introPage=intro_3.html.

[17] McPherson K., Steel C. M., Dixon J. M. (2000). ABC of breast diseases: breast cancer—epidemiology, risk factors, and genetics. *British Medical Journal*.

[18] Anderson K. N., Schwab R. B., Martinez M. E. (2014). Reproductive risk factors and breast cancer subtypes: a review of the literature. *Breast Cancer Research and Treatment*.

[19] Ritte R., Tikk K., Lukanova A. (2013). Reproductive factors and risk of hormone receptor positive and negative breast cancer: a cohort study. *BMC Cancer*.

[20] Yang X. R., Chang-Claude J., Goode E. L. (2011). Associations of breast cancer risk factors with tumor subtypes: a pooled analysis from the Breast Cancer Association Consortium studies. *Journal of the National Cancer Institute*.

[21] Ma H., Bernstein L., Ross R. K., Ursin G. (2006). Hormone-related risk factors for breast cancer in women under age 50 years by estrogen and progesterone receptor status: results from a case–control and a case–case comparison. *Breast Cancer Research*.

[22] Renehan A. G., Tyson M., Egger M., Heller R. F., Zwahlen M. (2008). Body-mass index and incidence of cancer: a systematic review and meta-analysis of prospective observational studies. *The Lancet*.

[23] Nichols H. B., Trentham-Dietz A., Love R. R. (2005). Differences in breast cancer risk factors by tumor marker subtypes among premenopausal Vietnamese and Chinese women. *Cancer Epidemiology, Biomarkers & Prevention*.

[24] Carey L. A., Perou C. M., Livasy C. A. (2006). Race, breast cancer subtypes, and survival in the Carolina Breast Cancer Study. *The Journal of the American Medical Association*.

[25] Yost K., Perkins C., Cohen R., Morris C., Wright W. (2001). Socioeconomic status and breast cancer incidence in California for different race/ethnic groups. *Cancer Causes & Control*.

[26] Braaten T., Weiderpass E., Kumle M., Adami H.-O., Lund E. (2004). Education and risk of breast cancer in the Norwegian-Swedish women's lifestyle and health cohort study. *International Journal of Cancer*.

[27] Love R. R., Dinh N. V., Ta V. T. (2010). Family history and BRCA mutations in Vietnamese breast cancer patients. *Clinical Genetics*.

[28] Nakamura S., Kwong A., Kim S.-W. (2016). Current status of the management of hereditary breast and ovarian cancer in Asia: first report by the Asian BRCA consortium. *Public Health Genomics*.

[29] Shinde S. S., Forman M. R., Kuerer H. M. (2010). Higher parity and shorter breastfeeding duration. *Cancer*.

[30] Gaudet M. M., Press M. F., Haile R. W. (2011). Risk factors by molecular subtypes of breast cancer across a population-based study of women 56 years or younger. *Breast Cancer Research and Treatment*.

